# Briumvi: a breakthrough in the treatment of relapsing multiple sclerosis: a review

**DOI:** 10.1097/MS9.0000000000001184

**Published:** 2023-08-16

**Authors:** Ayesha Azhar, Muskan Asim Taimuri, Shamat Fathi Shamat, Areeba Ikram, Sajjad Ali, Tehreem Ali, Yumna Khabir

**Affiliations:** a Department of Internal Medicine, Dow University of Health Sciences; b Department of Medicine, Ziauddin Medical University, Karachi, Pakistan; c Faculty of Medicine, National Ribat University, Khartoum, Sudan

**Keywords:** antibody, B cell therapy, drug, FDA, multiple sclerosis

## Abstract

Multiple sclerosis (MS) is a chronic systemic autoimmune disorder characterized by plaques of demyelination, autoimmune inflammation, and astrocytic gliosis. The primary cells involved in the pathophysiology of MS are T cells. However, B cells have recently been implicated in the pathophysiology of the disease. Therefore, researchers have been exploring B cell therapy as an alternative treatment option for MS. B cell therapy is based on the targeted depletion of CD20-positive B cells. Rituximab, ocrelizumab, and ofatumumab are anti-CD20 antibodies already approved. Briumvi, the fourth type of anti-CD20 antibody was approved by FDA in December 2022, for the treatment of relapsing types of MS, including relapsing-remitting multiple sclerosis, active secondary progressive multiple sclerosis, and clinically isolated syndromes after the drug was tested in two randomized, double-blind, phase III, ULTIMATE I, and II trials which compared Briumvi (ublituximab) with Aubiago (teriflunomide). Ublituximab was found to have a much lower annual relapse rate in the ULTIMATE II trials than teriflunomide. Briumvi is a chimeric recombinant IgG1 monoclonal antibody directed against human CD20 with potential antineoplastic activity. Its mechanism of action involves several distinct processes that collectively lead to the depletion of B cells and suppression of the immune response. The primary mode of action of Briumvi is its high-affinity binding to CD20. Infusion-related reactions are the most common side effects encountered following intravenous administration of ublituximab.

## Introduction

Highlights

- The lower annual relapse rate, higher potency, and shorter infusion time make Briumvi’s approval a significant step forward in the treatment of relapsing multiple sclerosis.
- The short infusion times of Briumvi could assist medical facilities with a high patient volume to treat more patients during a given period.
- More studies about the efficacy and safety of the drug are necessary to confirm the side effects of prolonged treatment.

Multiple sclerosis (MS) is a chronic autoimmune disorder characterized by plaques of demyelination, autoimmune inflammation, and astrocytic gliosis^1^. MS lesions can occur anywhere in the CNS, but certain regions of predilection include the supratentorial brain, particularly the frontal lobe and sub-lobar region^2^. The course of disease progression varies greatly amongst individuals, according to which MS is classified into three major types: relapsing-remitting multiple sclerosis (RRMS), secondary progressive multiple sclerosis (SPMS), and primary progressive multiple sclerosis (PPMS). In most cases, the disease initially follows a relapsing and remitting course. RRMS shows clear exacerbations of neurological symptoms known as relapses, followed by a stage of remission with no disease progression. SPMS often follows RRMS and is characterized by a deterioration of symptoms, with a reduction in relapses and no apparent signs of remission. In PPMS, symptoms progressively worsen without clearly defined relapses or remission periods^1^. Clinically, MS is diagnosed based on visually evoked potential tests, lesions demonstrated by MRI, and cerebrospinal fluid analysis^1^.

MS is a systemic disorder that can cause various symptoms, such as vertigo, double vision, tingling or numbness in the limbs, bowel disturbances, and cognitive impairment^1^. Consequently, the most common treatment options include medications to manage these symptoms, such as corticosteroids to reduce inflammation during exacerbations, muscle relaxants to reduce muscle spasms and stiffness, antidepressants, and analgesics^3^. Specific treatment options for relapsing forms of MS include disease-modifying therapies, which reduce exacerbations and slow down the progression of the disease by modulating the immune system^4^. Injectable disease-modifying therapies used for RRMS include beta-interferons (IFN-β) and glatiramer acetate, both of which reduce the overexpression of proinflammatory genes to slow down the progression of the disease^5^.

## Pathophysiology of MS

MS is a complex neurodegenerative disorder involving neuronal injury and inflammatory plaques, resulting from overactivation of the immune system, that causes immune cells to cross the blood-brain barrier^6^. The primary cells involved in the pathophysiology of MS are T cells; autoreactive T cells develop resistance to regulatory mechanisms, and antigens are presented to naïve T cells by antigen-presenting cells (APCs), which cause the phenotypic differentiation of T cells into CD 8+ T cells, and CD4+ Th1 and Th17 cells^6^. However, B cells have recently been implicated in the pathophysiology of the disease; B cells act as both APCs for the activation of T cells into inflammatory cells^7^ and are precursors of antibody-secreting plasma cells, which are involved in the demyelination of axons and formation of lesions in the CNS^7^. Furthermore, along with releasing proinflammatory cytokines, a subset of B cells, B2 cells, are also involved in the activation of regulatory T cells, which are anti-inflammatory^7^ and might be manipulated to control the progression of MS. Therefore, researchers have been exploring B cell therapy as an alternative treatment option for MS.

## B Cell Therapy for MS

B cell therapy is based on the targeted depletion of CD20-positive B cells, which aid in the propagation of MS by releasing proinflammatory cytokines and acting as APCs. Anti-CD20 monoclonal antibodies bind to specific epitopes on CD20, a transmembrane protein, which acts as a surface marker present during the differentiation of B cells. Rituximab, a chimeric monoclonal antibody, was the first anti-CD20 antibody developed and tested in phase I and a subsequent phase II trial on 439 patients with PPMS. While it was associated with a lower annualized relapse rate (ARR) and a reduction in the number of T2 and gadolinium-enhancing brain lesions, it was limited by its efficacy^8^.

Further progress led to the development of ocrelizumab, a humanized anti-CD20 IgG1 antibody administered intravenously and binds to a different epitope on CD20. Orcelizumab was tested in various trials, including two randomized phase III trials conducted over a period of 96 weeks, which demonstrated a lower ARR with ocrelizumab and a 94% decrease in gadolinium-enhancing lesions^8^. Ocrelizumab was approved by the US Food and Drug Administration (FDA) in 2017, but further trials were required to judge its efficacy and safety in young adults. Ofatumumab, the third anti-CD20 antibody, is fully humanized and binds to two different epitopes on CD20, the small and large extracellular loops. It was tested to have a 99% decrease in the development of new T1 and T2 gadolinium-enhancing lesions, along with a lower relapse rate^8^. However, several adverse effects were reported with ofatumumab, such as thrombocytopenia, anemia, and infusion reactions, casting doubts on its use as a broad-scale treatment for various forms of MS^7^.

On 28 December 2022, the US Food and Drug Administration (FDA) approved Briumvi, the third type of anti-CD20 B cell antibody, for the treatment of relapsing types of MS, including RRMS, active SPMS, and clinically isolated syndrome^9^.

## Mechanism of Action of ublituximab (Briumvi)

Briumvi (ublituximab) is a glycoengineered chimeric IgG1 antibody that targets a specific epitope on the B cell antigen CD20, distinct from the ones targeted by rituximab, ocrelizumab, and oftamumab^10^, and promotes antibody-dependent cellular cytolysis (ADCC) of the B cell by natural killer (NK) cells^10^. Ublituximab’s affinity for CD16A, a membrane receptor involved in the activation of NK cells, is increased by the reduction in frucose concentration after glycoengineering^11^, which increases its antibody-dependent cellular cytolysis activity by 25–30 times^10^. In addition to conserving CD20-negative plasma cells, this targeted depletion of CD20-positive immature and mature B cells is associated with a decrease in the frequency of new and progressing brain lesions^7^.

## Clinical Efficacy

### Ublituximab versus teriflunomide

Briumvi was approved after the drug was tested in two randomized, double-blind, phase III, ULTIMATE I, and II trials conducted on 1094 patients with RRMS^10^. Patients were randomly divided in an equal ratio to receive IV ublituximab and an oral placebo OR oral teriflunomide and an IV placebo. The outcome was to assess the ARR in the two groups. The study compared Briumvi (ublituximab) with Aubiago (teriflunomide) over 96 weeks and found ublituximab to have a much lower ARR in the ULTIMATE II trials than teriflunomide, at 0.09 as opposed to 0.18^10^. The trials also reported that the probability of development of T1 and T2 gadolinium-enhancing lesions was reduced in patients treated with ublituximab^10^. However, the comparison also revealed that at 12 weeks, 5.2% of the ublituximab group showed worsening of disability as compared to 5.9% of participants in the teriflunomide group, which was reduced to 3.3 and 4.8%, respectively, by week 24^10^. Affecting nearly half of the patients treated with ublituximab, the most insidious of the outcomes were infusion-related reactions (IRR) and severe infections, which were reported in 5% of the patients, as compared to 2.9% of those on teriflunomide^10^. The mean number of new and hyperintense lesions was also more significant in the teriflunomide group. The average count in CD19+ B cells at the end of the double-blind trial was reduced by 97% in the ublituximab group and 18% in those on teriflunomide. However, the percent change in brain volume was not reported to be considered significant between the two groups^10^.

### Ofatumumab versus teriflunomide

In another trial of a similar set-up, teriflunomide was compared with ofatumumab in efficacy^12^. The results depicted a similar picture as the trial evaluating teriflunomide and ublituximab. In trial 1, ofatumumab had an ARR of 0.11 and teriflunomide 0.22, which in trial two was found to be 0.10 and 0.25, respectively. Disability worsening outcomes confirmed at 3 months were reportedly seen in 10.9% of patients in the ofatumumab group and 15% in the teriflunomide group, but at 6 months, the number was reduced to be seen in 8.1 and 12% of participants in the respective groups. Hence, disability worsening outcomes confirmed at 3 months and 6 months were lower in the ofatumumab group than in teriflunomide. However, as was reported for ublituximab, ofatumumab reported IRR in 20.2% of the group and serious infections in 2.5% of the population as compared to 15.0% IRR and 1.8% seriously infected patients treated with teriflunomide^12^.

### Ublituximab versus ofatumumab

Among the MS patients, ofatumumab and ublituximab reported better ARR in their individualized trials with teriflunomide. The drugs differ in their pharmacokinetics and route of administration, which alter the patient’s response significantly. Ublituximab is given intravenously, whereas ofatumumab is administered subcutaneously, reflecting the rapid bioavailability of ublituximab; however, a slower absorption abrogates side effects, as is the case with ofatumumab^13^. As mentioned previously, disability worsening outcomes for ofatumumab were confirmed to be lower than that of teriflunomide^13^; however, the ULTIMATE trials revealed no significant reduction in the risk of disability worsening when comparing ublituximab and teriflunomide^12^. Thus, studies comparing ublituximab and ofatumumab are needed to unfold the relative efficacy of these two drugs.

### Safety and tolerability

IRRs are the most commonly encountered complication of ublituximab administration. These present within 24 h of the first dose administration with symptoms ranging from mild to moderate severity, usually subsiding with subsequent doses^13^. Severe infections can ensue as a side effect of ublituximab. Most commonly encountered are upper and lower respiratory tract infections and herpes-virus-associated infections^9^. Prolonged B cell depletion following anti-CD20 antibodies is the precipitating factor in the pathogenesis of infections. The trial data reflects the need to control these infections, as reported in nearly 5% of patients treated with ublituximab^13^. Ublituximab is generally a well-tolerated drug with great bioavailability following its intravenously administration; no fatal side effects are reportedly associated except for mild pain in the extremities, insomnia, and fatigue^9^. However, its rapid action might present a similar surge of side effects. The formation of antidrug antibodies ADAs is another challenge that aligns with using these monoclonal antibodies. Fully human monoclonal antibodies like ofatumumab are the least immunogenic^13^. However, more statistical data are required following the use of ublituximab for reports on its immunogenicity. Briumvi is contraindicated in pregnancy and prior hepatitis B infection^9^.

## Conclusion

The lower ARR, higher potency, and shorter infusion time make Briumvi’s approval a significant step forward in the treatment of relapsing MS, a condition affecting millions worldwide. The short infusion times of Briumvi could assist medical facilities with a high patient volume to treat more patients during a given period. This could be crucial in treating MS in middle-income and lower-middle-income nations with a shortage of medical professionals, limited access to resources, and underdeveloped healthcare infrastructure. However, considering the potential severity of the side effects associated with Briumvi, healthcare workers must approach its administration responsibly. Specialized physicians with ample experience in diagnosing and treating neurological conditions and access to appropriate medical support to manage severe reactions such as IRR and immunosuppression should be entrusted with initiating this drug. More studies about the efficacy and safety of the drug are necessary to confirm the side effects of prolonged treatment, such as progressive multifocal leukoencephalopathy or the safety of immunization with live or live-attenuated vaccines during or following administration of Briumvi^9^. Lastly, improved patient support programs and competitive pricing are essential to compete with other anti-CD20 B cell antibodies and gain a large patient share in the MS market.

## Ethics approval and consent to participate

Not applicable.

## Consent

Not applicable.

## Sources of funding

This research did not receive any specific grant from funding agencies in the public, commercial, or not-for-profit sectors.

## Conflicts of interest disclosure

The authors declare that they have no conflicts of interest.

## Author contribution

A.A.: writing – original draft, writing – review and editing; M.A.T.: writing – original draft, writing – review and editing; A.I.: writing – original draft; S.A.: writing – review and editing; T.A.: writing – review and editing; Y.K.: writing – original draft; S.F.S.: writing – review and editing.

## Research registration unique identifying number (UIN)

1. Name of the registry: not applicable.
2. Unique identifying number or registration ID: not applicable.
3. Hyperlink to your specific registration (must be publicly accessible and will be checked): not applicable.

## Guarantor

Ayesha Azhar, Muskan Asim Taimuri, Yumna Khabir.

## Provenance and peer review

Not commissioned, externally peer reviewed.

## Availability of data and materials

Data sharing is not applicable to this article as no datasets were generated or analyzed during the current study.
